# Brief parenting intervention for parents of NICU graduates: a randomized, clinical trial of Primary Care Triple P

**DOI:** 10.1186/1471-2431-13-69

**Published:** 2013-05-07

**Authors:** Renske Schappin, Lex Wijnroks, Monica Uniken Venema, Barbara Wijnberg-Williams, Ravian Veenstra, Corine Koopman-Esseboom, Susanne Mulder-De Tollenaer, Ingeborg van der Tweel, Marian Jongmans

**Affiliations:** 1 Department of Medical Psychology and Social Work, Wilhelmina Children’s Hospital, UMC Utrecht, Utrecht, The Netherlands; 2 Department of Special Education, Faculty of Social and Behavioral Sciences, Utrecht University, Utrecht, The Netherlands; 3 Department of Medical Psychology, Isala Clinics, Zwolle, The Netherlands; 4 Department of Neonatology, Wilhelmina Children’s Hospital, UMC Utrecht, Utrecht, The Netherlands; 5 Department of Neonatology, Isala Clinics, Zwolle, The Netherlands; 6 Julius Center for Health Sciences and Primary Care, UMC Utrecht, Utrecht, The Netherlands

**Keywords:** Primary Care Triple P, Parenting intervention, Preterm birth, Perinatal asphyxia, RCT

## Abstract

**Background:**

Preterm-born or asphyxiated term-born children who received neonatal intensive care show more emotional and behavioral problems than term-born children without a medical condition. It is uncertain whether regular parenting intervention programs to which the parents of these children are usually referred, are effective in reducing child problem behavior in this specific population. Our objective was to investigate whether a regular, brief parenting intervention, Primary Care Triple P, is effective in decreasing emotional and behavioral problems in preterm-born or asphyxiated term-born preschoolers.

**Methods:**

For this pragmatic, open randomized clinical trial, participants were recruited from a cohort of infants admitted to the neonatal intensive care units (NICU) of two Dutch hospitals. Children born with a gestational age <32 weeks or birth weight <1500 g and children born at a gestational age 37–42 weeks with perinatal asphyxia were included. After screening for a *t*-score ≥60 on the Child Behavior Checklist (CBCL), children were randomly assigned to Primary Care Triple P (n = 34) or a wait-list control group (n = 33). The primary outcome was child emotional and behavioral problems reported by parents on the CBCL, 6 months after the start of the trial.

**Results:**

There was no effect of the intervention on the CBCL at the trial endpoint (*t*_64_ = 0.54, *P* = .30). On secondary measurements of child problem behavior, parenting style, parenting stress, and parent perceived child vulnerability, groups either did not differ significantly or the intervention group showed more problems. In both the intervention and control group there was a significant decrease in emotional and behavioral problems during the trial.

**Conclusions:**

Primary Care Triple P, a brief parenting intervention, is not effective in reducing child emotional and behavioral problems in preterm-born children or term-born children with perinatal asphyxia.

**Trial registration:**

Netherlands National Trial Register (NTR): NTR2179

## Background

Preterm-born or asphyxiated term-born children who receive neonatal intensive care show more emotional and behavioral problems than term-born children without a medical condition [1-4]. These children are two major patient groups in the neonatal intensive care unit (NICU), and have a prevalence of behavior problems of 20%, versus approximately 10% in healthy term-born children [3,5]. Furthermore, having a child in the NICU burdens parenting, not only during the acute phase of illness but also in the years thereafter [6]. Transactional theories on the development of behavior problems in preterm-born children suggest that the interplay between parents’ preexisting personality and family factors, prenatal experiences, and emotional distress during the NICU period, alters parents’ perception of their child and their parenting style [7]. Parents may inadequately perceive their child as extra vulnerable even up to 6 years after birth [8,9], and employ a parenting style that is characterized by overprotection and inconsistent discipline [10]. In combination with the neurological vulnerability of the child, these altered parenting practices may negatively impact the behavior of the child [11]. Therefore, there is a need for interventions that support these parents in reducing their child’s problem behavior and emphasize their competence as parents.

Existing parenting interventions for preterm-born and asphyxiated term-born children are aimed at preventing emotional and behavioral problems and take place during the neonatal period. Although the effects of these early interventions are positive, they are usually short-term [12,13]. Since problem behavior generally surfaces around two years of age in preterm-born children [14], there is a need for specific, problem-based parenting interventions for parents of NICU graduates at preschool age. Current practice is that parents of preschool-aged NICU graduates are referred to regular parenting interventions. However, given the impact of NICU admission on families [7], it is uncertain whether regular parenting intervention programs are effective in families with a preterm-born or asphyxiated term-born child. Therefore, in these families, we investigated the effectiveness of a widely-used brief parenting intervention called Primary Care Triple P. Triple P is a stepped-care system of parenting interventions that aims to reduce child problem behavior by improving the competences and the self-reliance of parents in terms of their parenting behavior, in parents of children between 0 and 12 years old [15]. A brief version of Triple P was chosen because it fitted the problems reported by parents during regular clinical follow-up. Furthermore, at the time our trial was designed, several studies had demonstrated the effectiveness of Primary Care Triple P in non-clinical populations [16,17].

Consequently, our primary question was whether a regular, brief parenting intervention like Primary Care Triple P would be effective in decreasing emotional and behavioral problems in preterm-born or asphyxiated term-born preschoolers. Secondary questions were whether Primary Care Triple P would be effective in decreasing parenting stress, would alter parenting skills, and would decrease parent-perceived child vulnerability. To investigate these questions, we conducted a pragmatic, open randomized clinical trial (RCT) in which parents of 2- to 5-year-old preterm-born or asphyxiated term-born children received Primary Care Triple P.

## Methods

### Study design

The study was a pragmatic, open RCT, with a wait-list control group. Two Dutch medical centers with a NICU participated in the study: the Wilhelmina Children’s Hospital (Utrecht) and the Isala Clinics (Zwolle). Approval for the study was obtained from the research ethics committees for both centers.

### Participants

Participants were recruited by mail from a cohort of infants born between September 2004 and October 2007, and subsequently admitted to the NICUs of the two participating centers. Children born with gestational ages <32 weeks or birth weights <1500 g were eligible for the screening phase of this study, together with children born at a gestational age of 37–42 weeks showing clinical signs of perinatal asphyxia (Apgar score <5 at 10 minutes, prolonged resuscitation, acidosis, and base deficits). We excluded children with cognitive and/or motor impairments (defined as a developmental quotient <70 and/or a Gross Motor Function Classification System score >3 [18]), children with parents who did not speak Dutch, and children from families that had received a parenting intervention in the last 6 months.

**Figure 1:**
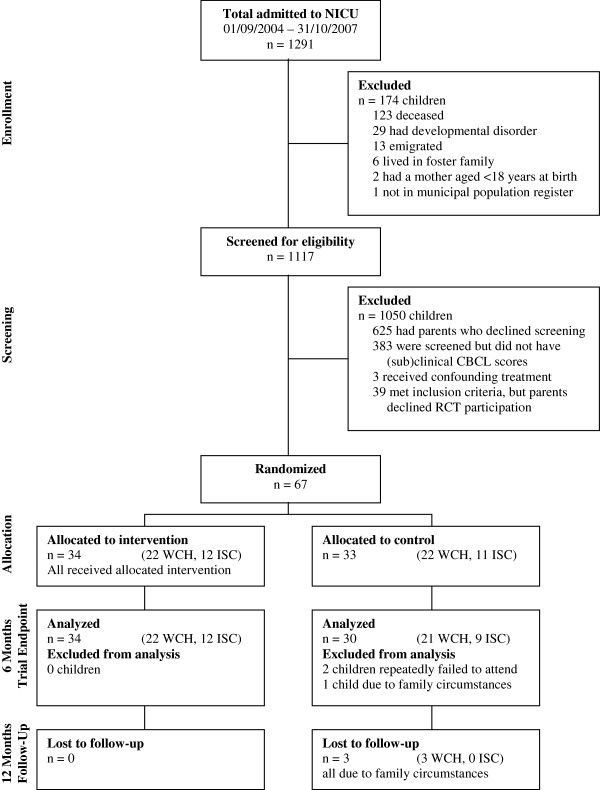
**Participant flow through screening, trial, and follow-up.** Abbreviations: WCH, Wilhelmina Children’s Hospital; ISC, Isala Clinics.

Eligible children and their families that consented were screened for children’s emotional and behavioral problems. Children whose parents reported a *t*-score ≥60 on the internal, external, or total problem scale of the Child Behavior Checklist (CBCL) [19], were eligible for randomization. Originally, we had also planned to use the Dutch version of the Parenting Stress Index – Short Form (PSI-SF; Nijmeegse Ouderlijke Stress Index-Kort) as a screening tool [20], but although the PSI-SF and CBCL scores were correlated (*r* = .69, *P* < .001 [n = 491]), it became apparent that almost none of the children’s parents met the inclusion criterion on the PSI-SF (a raw total stress score ≥121). Therefore, during inclusion the PSI-SF was abandoned as a criterion, and children from parents formerly excluded based on their PSI-SF scores were once again invited to participate. Because the low levels of parenting stress were an interesting and surprising phenomenon, we investigated this further by conducting a meta-analysis of studies examining parenting stress in parents of preterm infants [21]. Parents’ informed consent was obtained separately for the screening and randomized phase of the study. See Figure 1 for participant flow and Additional file 1 for the CONSORT form.

### Randomization

Random assignment was stratified for each center and, due to the nature of the intervention, when twins were included both siblings received the same treatment. Open allocation of children to either the intervention or control group at a ratio of 1:1 was performed by the first author, according to computer-generated random permuted blocks of 6 [22]. After the trial endpoint at 6 months, children and their parents in both the intervention and the control group received appropriate (additional) psychological treatment when needed. Therefore, at the 12-month follow-up, groups were analyzed according to intention-to-treat, but allocation to treatment was no longer random.

### Intervention

Primary Care Triple P is a brief parenting intervention that consists of 4 sessions involving active skills training for parents of children with mild to moderate emotional or behavioral problems. Sessions took place in one of the two participating hospitals once a week, with a break of 3 weeks before the fourth session, and both parents were encouraged to attend sessions. The main objective of Triple P is to reduce child problem behavior by improving parent competence and self-reliance in terms of parenting [15].

The Primary Care Triple P training was provided by one experienced social worker, two registered healthcare psychologists, and two registered clinical psychologists. They received three-and-a-half-days of training in Primary Care and Standard Triple P and passed an individual examination and accreditation test to become licensed Triple P practitioners. Peer supervision between these professionals and the first author conducted at least once a month assured adherence to the intervention protocol.

During the first 6 months of the study, the intervention group did not receive any intervention other than Primary Care Triple P. At 6 months after the start of the study, intervention group children and their parents requiring additional support received further treatment from their assigned Triple P professional or were referred to other health care providers if necessary. We ensured that the control group children and their parents did not receive any intervention until 6 months after the start of the study. At this time, control group children and their parents could choose to receive Primary Care Triple P or another type of psychological treatment suited to their problems, or to be referred to other health care professionals.

### Outcome measures

Parents completed questionnaires at baseline, 6 months, and 12 months, all at home. The time-point of 6 months was the direct outcome of this trial. At 12 months, a follow-up measure was conducted. Immediately after their last Primary Care Triple P session, the intervention group parents completed the Client Satisfaction Questionnaire (CSQ; parent evaluation of the program) in the hospital [19,23]. Neonatal variables were obtained from the child’s medical records. Family background variables were assessed at baseline.

The primary outcomes were child emotional and behavioral problems, assessed with the preschool CBCL [19], which yields standardized *t*-scores for internalizing, externalizing, and total problem behavior. The CBCL is the most commonly-used questionnaire to evaluate child behavior and enables Primary Care Triple P effects to be compared with those of other interventions. All other outcomes were secondary and also parent-reported, except for the preschool Teacher Report Form (TRF) [19], which is an assessment of child emotional and behavioral problems completed by the child’s daycare provider or teacher. The TRF consists of the same questionnaires and scale scores as the CBCL. Child emotional and behavioral problems were also assessed with the Eyberg Child Behavior Inventory (ECBI) [24], a 36-item questionnaire for children aged 2 to 16 years which yields standardized *t*-scores for the intensity and frequency of problem behavior. The ECBI is often used to estimate the effect of Triple P programs, and therefore allows our results to be compared with other studies on Primary Care Triple P. Parenting stress was measured with the Dutch version of the PSI [20], which returns a total scaled score of 123 items measuring parenting stress in parents of children aged 1 month to 12 years. The Child Rearing Practices Report (CRPR) was used to assess parenting styles [25]. This is a 91-item questionnaire measuring parent attitudes toward parenting and yields two scaled scores: one for nurturing and one for restrictive parenting styles. Finally, parents reported on the perceived vulnerability of their child with the Vulnerable Child Scale (VCS) [26], a 16-item questionnaire which describes parents’ health concerns about their children, yielding a total vulnerability score. The VCS and the CRPR nurture scale are the only scales on which high scores indicate favorable outcomes.

### Sample size calculation

Sample size was based on the possibility to detect whether the intervention group scored 5 points lower on the CBCL total problem *t*-score than the control group at the 6-month measurement point, assuming no decline in the *t*-score of the wait-list control group. Because a decrease in problems was expected, a one-sided 5% significance level was chosen. With a power of 80%, an attrition rate of 10%, and a standard deviation of 7.1 based on preliminary findings, we calculated that we needed a minimum sample size of 32 children per treatment arm.

### Statistical analyses

Linear mixed models were used to estimate the effects of Primary Care Triple P on the primary and secondary outcomes. Within linear mixed models, changes from baseline to successive time-points in the intervention group are compared to changes in the same time-period for the control group. In this study, differences between the intervention and control group, development of problems in the randomized phase (baseline to 6 months) and non-randomized phase (6 months to 12 months) of the study, and the interaction between group and time were investigated. Models were adjusted for the medical centers that served as a stratification variable, although due to the small number of centers only fixed effects for the medical center variable were included in the model [27]. Group, time-point, medical center, and the interaction between group and time-point were included as fixed effects in the model. Intercept and slope of the change over time were included as random effects. Including these random effects in the model enables variation in individual developmental trajectories. Covariance structures are specified in the results tables. Model fit was assessed using IBM SPSS version 20 [28], with REML estimation. Missing values were not imputed. All available data were used, with analyses based on the principle of intention-to-treat.

**Table 1:** **Baseline characteristics of children and parents participating in the intervention and control group**^**a**^

	**Intervention**	**Control**
	**(n = 34)**	**(n = 33)**
**Child characteristics**		
Age, mean (SD), mo	45.6 (10.0)	43.6 (10.7)
Males	16 (47%)	24 (73%)
Twins^b^	5 (15%)	4 (12%)
BW, mean (SD), g	1477.1 (849.3)	1626.7 (876.3)
GA, mean (SD), wk	30.5 (4.2)	30.8 (4.5)
Abnormal cerebral ultrasound		
IVH grade 1-2	31 (91%)	31 (94%)
IVH grade 3-4	3 (9%)	2 (6%)
Perinatal asphyxia (term-born)	3 (9%)	5 (15%)
NICU stay, mean (SD), d	21.4 (20.1)	18.6 (16.2)
**Family characteristics**		
Maternal age, mean (SD), y	34.1 (5.5)	32.2 (5.2)
Maternal ethnicity		
European	33 (97%)	33 (100%)
North-African	1 (3%)	0
Firstborn child	26 (77%)	27 (82%)
Maternal education, mean (SD), y	14.5 (2.1)	14.7 (2.0)
Paternal education, mean (SD), y	14.2 (2.8)	14.8 (2.4)
Family situation		
Nuclear family	32 (94%)	30 (91%)
Stepfamily	1 (3%)	0
Single parent family	1 (3%)	3 (9%)
Questionnaires completed by mother	32 (94%)	33 (100%)

Abbreviations: BW, birth weight; GA, gestational age; IVH, intraventricular hemorrhage.

^a^Data are presented as number (percentage) unless otherwise specified.

^b^Including 1 set of triplets in the control group.

**Table 2:** **Raw means and standard deviations of primary and secondary outcomes at baseline, 6-month, and 12-month follow-up**^**a**^

		**Baseline**				**6-month trial endpoint**				**12-month follow-up**			
**Outcome**	**Clinical cutoff score**	**n**	**Intervention**	**n**	**Control**	**n**	**Intervention**	**n**	**Control**	**n**	**Intervention**	**n**	**Control**
CBCL	≥60	34	61.7 (6.5)	33	61.0 (4.9)	31	56.6 (7.0)	28	57.1 (8.3)	30	56.8 (8.3)	26	55.5 (8.1)
TRF	≥60	31	53.7 (7.7)	30	51.7 (8.0)	31	54.7 (7.4)	26	50.1 (9.3)	25	54.4 (8.0)	25	50.9 (8.4)
ECBI intensity	≥60	32	63.0 (6.7)	32	59.9 (6.5)	33	59.8 (7.3)	30	58.2 (6.7)	30	59.4 (7.7)	26	58.3 (5.6)
ECBI frequency	≥60	32	59.3 (8.7)	32	53.3 (9.5)	33	53.0 (9.9)	30	49.5 (9.1)	30	52.2 (9.2)	26	46.4 (6.1)
PSI	n.a.	33	5.3 (1.2)	32	5.2 (0.9)	33	4.7 (1.3)	30	4.8 (1.2)	29	5.0 (1.2)	26	4.7 (1.1)
CRPR restrictive	n.a.	32	4.0 (0.6)	32	3.9 (0.6)	31	4.0 (0.5)	30	4.0 (0.4)	30	4.0 (0.6)	26	4.0 (0.5)
CRPR nurture	n.a.	32	1.8 (0.4)	32	1.8 (0.4)	31	1.8 (0.3)	30	1.8 (0.4)	30	1.8 (0.3)	26	1.8 (0.3)
VCS	n.a.	32	50.4 (5.3)	32	49.9 (6.7)	33	52.7 (4.8)	30	51.0 (6.7)	30	51.8 (4.9)	26	53.0 (5.8)

Abbreviations: CBCL, Child Behavior Checklist; TRF, Teacher Report Form; ECBI, Eyberg Child Behavior Inventory; PSI, Parenting Stress Index; CRPR, Child Rearing Practices Report; VCS, Vulnerable Child Scale.

^a^Data are means (SD).

## Results

### Participants

Between May 2009 and March 2010, we recruited parents of 2- to 5-year-old children who had been admitted to the NICUs of the Wilhelmina Children’s Hospital and the Isala Clinics. Because of infant mortality or known ineligibility for screening, 174 children were excluded beforehand. The 1117 remaining children were all approached for screening, and 492 children’s parents consented. Of these 492 children, 106 met inclusion criteria for randomization, and 67 children’s parents consented to participate in the randomized trial. The children participating in the randomized clinical trial were randomly allocated to the intervention (n = 34) or control group (n = 33). Questionnaires were all completed by mothers, except for 2 in the intervention group that were completed by fathers. During the first 6 months of the trial, 3 children dropped out of the control group, 1 child’s parents were not able to take leave from work, and 2 children’s parents repeatedly failed to complete questionnaires. By the 12-month follow-up, 3 more children had dropped out, respectively due to emigration, a mother’s second complicated pregnancy, and severe illness in the family. In the control group, 17 children and their parents received an intervention after 6 months and, in the intervention group, 6 children and their parents received an additional intervention. In both groups, depending on the child’s or parents’ problems, interventions could range from a one-hour session with a psychologist to 4 sessions of Primary Care Triple P, to referral to a child psychiatrist.

Baseline characteristics of children and parents are presented in Table 1. There were no significant differences in demographic and neonatal characteristics between the total cohort and the RCT participants. We did not test for baseline differences between the intervention and control group. Since there was random allocation of participants to study groups, all differences between groups are coincidental.

### Primary child emotional and behavioral problems outcomes

For all outcomes, raw data are presented in Table 2 and the analysis of the 6-month trial endpoint is presented in Table 3. For the primary outcome of parent reported emotional and behavioral problems on the CBCL, there was no significant difference between the Primary Care Triple P intervention group and the wait-list control group at the 6-month trial endpoint. In both groups there was a significant decrease in emotional and behavioral problems from baseline onwards. Although emotional and behavioral problems decreased for the total group of children, there was variation in the individual developmental trajectories of CBCL scores from baseline to the trial endpoint.

**Table 3:** **Estimated fixed and random effects for primary and secondary outcomes from baseline to 6-month trial endpoint**^**a**^

			**Fixed effects**						**Random effects**			
**Outcome**	**Score range**	**n**	**Intervention**	***P***	**Time**	***P***	Intervention*time	***P***	**Intercept**	***P***	**Slope**	***P***
CBCL	0-100	67	0.77 (1.44)	.30	-0.44 (0.18)	.01	-0.15 (0.25)	.28	8.75 (5.99)^b^	.07	0.31 (0.16)	.03
TRF	0-100	65	2.53 (1.99)	.11	-0.31 (0.28)	.14	0.54 (0.39)	.08	32.36 (9.65)^b^	<.001	0.22 (0.49)	.33
ECBI intensity	33-94	65	3.41 (1.67)	.02	-0.34 (0.16)	.02	-0.26 (0.22)	.12	34.75 (7.34)^b^	<.001	0.14 (0.26)	.29
ECBI frequency	41-88	65	6.56 (2.33)	<.005	-0.66 (0.25)	<.005	-0.56 (0.34)	.06	60.99 (13.92)^b^	<.001	0.22 (0.56)	.35
PSI	1-6	66	0.11 (0.27)	.34	-0.08 (0.03)	<.005	-0.02 (0.04)	.29	1.04 (0.22)^b^	<.001	0.01 (0.01)	.03
CRPR restrictive	0-6	65	0.11 (0.15)	.23	0.01 (0.01)	.13	-0.01 (0.02)	.29	0.34 (12.77)^d^	.49	0.00 (0.84)	.50
CRPR nurture	0-6	65	Model does not fit the data.									
VCS	16-64	65	0.68 (1.45)	.32	0.20 (0.27)	.23	0.23 (0.38)	.27	0.06 (0.29)^c^	.42	0.06 (0.29)	.42

Abbreviations: CBCL, Child Behavior Checklist; TRF, Teacher Report Form; ECBI, Eyberg Child Behavior Inventory; PSI, Parenting Stress Index; CRPR, Child Rearing Practices Report; VCS, Vulnerable Child Scale.

^a^Data are estimates (SE). All tests are one-sided.

^b^Random effects covariance structure is diagonal; repeated measures covariance structure is scaled identity.

^c^Random effects covariance structure is scaled identity; repeated measures covariance structure is scaled identity.

^d^Random effects covariance structure is scaled identity; repeated measures covariance structure is first-order autoregressive with heterogeneous variances.

### Secondary child emotional and behavioral problems outcomes

There was no significant difference between the Primary Care Triple P intervention group and the control group on emotional and behavioral problems reported by the teacher on the TRF at the 6-month trial endpoint. However, there was variation between individual children in the intercept of the TRF model. Contrary to parent reported emotional and behavioral problems, there were no significant changes in teacher reported emotional and behavioral problems in either group.

Unexpectedly, on both the intensity and frequency scale of the ECBI the Primary Care Triple P intervention group had significantly more intense and more frequent problems than the wait-list control group at the 6-month trial endpoint. Both ECBI models contained variation between individual children in the models’ intercepts. The intensity and frequency of problems decreased up to the trial endpoint for both the intervention and control group.

### Parenting outcomes

Parenting stress measured with the PSI did not differ between the Primary Care Triple P intervention and the wait-list control group at the 6-month trial endpoint. Stress significantly decreased in both groups from baseline to the trial endpoint. Individual children’s parents varied in both their intercept and their developmental trajectory of parenting stress. Regarding both the VCS and CRPR restrictive scale questionnaires, the intervention and control group did not differ on either parent reported vulnerability of their child or restrictive parenting at the 6-month trial endpoint. Both VCS and CRPR restrictive scores were stable over time, and there was no variation between individual children in the VCS or the CRPR restrictive model. Unfortunately, the model of the CRPR nurturing parenting style did not fit the data, irrespectively of covariance structures. This is probably due to a lack of variance in the CRPR nurturing outcomes. Parents in the intervention group reported their satisfaction with Primary Care Triple P on the CSQ with a mean of 6.2 (SD = 0.4) on a scale of 1 to 7.

### 12-month follow-up outcomes

The analysis of the 12-month follow-up outcomes is presented in Table 4. Due to the possibility of (additional) psychological treatment in both the intervention and control group after the trial endpoint at 6 months, allocation was no longer random during follow-up and results should be interpreted with care. Regarding the parent reported emotional and behavioral problems on the CBCL, none of the effects were significant. Individual children only varied in their model intercepts. On the TRF, the Primary Care Triple P intervention group and the wait-list control group differed significantly at the 12-month follow-up, with more problems for the intervention group. Furthermore, individual children varied in their intercept and had different developmental trajectories of teacher reported emotional and behavioral problems. On the PSI, both ECBI scales, and both CRPR scales, none of the effects were significant except for individual variation between children in the intercept and variation between developmental trajectories in these models (except for variation in only the intercept for the CRPR restrictive model). The VCS revealed a significant interaction between the Primary Care Triple P intervention and time, with increasing parent perceived child vulnerability for the intervention group, and decreasing perceived vulnerability for the control group between the 6-month trial endpoint and the 12-month follow-up. Furthermore, the VCS model contained variation between individual children in both the intercept and the developmental trajectory of parent perceived vulnerability.

**Table 4:** **Estimated fixed and random effects for primary and secondary outcomes from 6-month trial endpoint to 12-month follow-up**^**a**^

			**Fixed effects**						**Random effects**			
**Outcome**	**Score range**	**n**	**Intervention**	***P***	**Time**	***P***	Intervention*time	***P***	**Intercept**	***P***	**Slope**	***P***
CBCL	0-100	63	-2.12 (4.00)	.30	-0.23 (0.24)	.17	0.20 (0.33)	.27	28.23 (15.29)^b^	.03	0.08 (0.10)	.21
TRF	0-100	60	5.84 (3.40)	.05	0.06 (0.31)	.42	-0.19 (0.44)	.33	0.45 (0.14)^c^	<.005	0.45 (0.14)	<.005
ECBI intensity	33-94	64	2.44 (2.87)	.20	0.07 (0.26)	.40	-0.14 (0.36)	.35	0.28 (0.09)^c^	<.005	0.28 (0.09)	<.005
ECBI frequency	41-88	64	1.31 (3.88)	.37	-0.52 (0.35)	.07	0.39 (0.48)	.21	0.40 (0.15)^c^	<.005	0.40 (0.15)	<.005
PSI	1-6	63	-0.36 (0.52)	.25	-0.01 (0.05)	.39	0.05 (0.07)	.23	0.01 (0.00)^c^	<.005	0.01 (0.00)	<.005
CRPR restrictive	0-6	63	0.10 (0.16)	.26	0.01 (0.01)	.28	-0.01 (0.02)	.22	0.16 (0.04)^b^	<.001	0.00 (0.00)	.05
CRPR nurture	0-6	63	0.01 (0.15)	.48	0.00 (0.01)	.43	0.00 (0.02)	.45	0.00 (0.00)^c^	.01	0.00 (0.00)	.01
VCS	16-64	64	4.49 (2.13)	.02	0.33 (0.20)	.05	-0.47 (0.28)	.05	0.23 (0.07)^c^	<.001	0.23 (0.07)	<.001

Abbreviations: CBCL, Child Behavior Checklist; TRF, Teacher Report Form; ECBI, Eyberg Child Behavior Inventory; PSI, Parenting Stress Index; CRPR, Child Rearing Practices Report; VCS, Vulnerable Child Scale.

^a^Data are estimates (SE). All tests are one-sided.

^b^Random effects covariance structure is diagonal; repeated measures covariance structure is scaled identity.

^c^Random effects covariance structure is scaled identity; repeated measures covariance structure is scaled identity.

## Discussion

Primary Care Triple P was not effective in reducing child emotional and behavioral problems in preschool-aged preterm-born children or term-born children with perinatal asphyxia. Both the Primary Care Triple P group and the control group showed a significant decrease in problem behavior from baseline to the 6-month trial endpoint. Although there was much individual variation in children’s developmental trajectories, this was present in both groups and therefore did not alter the interpretation of the intervention effect. Other measures at the 6-month trial endpoint revealed no significant differences between the intervention and the wait-list control group, although parenting stress had decreased in both groups. At the 12-month follow-up, most measures showed no group differences or changes in behaviors.

Our finding that Primary Care Triple P is not effective in reducing problem behavior in NICU graduates could be due to the specific characteristics of our population. After all, the study was specifically designed to investigate the effectiveness of Primary Care Triple P in NICU graduates, assuming this version of Triple P to be effective for healthy term-born children. Given the specific parenting practices and parenting problems of parents of preschool-aged NICU graduates, such as inconsistent discipline which may lead to temper tantrums, a brief parenting program like Primary Care Triple P seemed indicated. However, this may have overlooked that a significant amount of attention should be paid to the emotional mechanisms behind these parents’ parenting practices. Feelings of guilt on behalf of the mother, lingering thoughts about the NICU period, and perceptions of vulnerability may need to be properly addressed before practical solutions find ground. This also suggests that it is possible that although Primary Care Triple P was not effective, more intensive versions of Triple P that consist of more sessions, such as Standard Triple P could have been effective.

Another possible explanation for our findings is a general lack of effectiveness of the Triple P program. Recently published independent research on Primary Care Triple P and Triple P in general questions the effectiveness of Triple P and suggests that it may not be as effective as was reported a few years ago. Currently, there are in total 5 published peer-reviewed publications evaluating Primary Care Triple P, of which 3 studies included a control group, but none included clinical populations [16,17,29-31]. The largest study employed a quasi-experimental design and found no significant differences between Primary Care Triple P and care-as-usual in terms of parenting stress, parenting practices, and family functioning [29]. Measures of child behavior were not included in this study. Another quasi-experimental study that compared Primary Care Triple P to care-as-usual did find significantly higher levels of parental competence and more positive parenting in the Primary Care Triple P group, but no significant differences between groups in terms of child emotional and behavioral problems [30]. The only study that used a wait-list control group in a quasi-experimental design was a developer-led study that was published before the start of our trial, and concluded significant differences existed between groups in terms of measures of child emotional and behavioral problems. The Primary Care Triple P group also had less dysfunctional parenting styles and less parental anxiety and depression. Nonetheless, groups did not differ with regards to observations of parent child interactions [16]. Compared with the two independent studies, the developer-led study reported the most positive results on the effect of Primary Care Triple P. This is in line with findings of a recent meta-analysis on Triple P in general, that independent researchers generally report smaller or non-existent effects compared to those seen in developer-led studies [32].

The general decrease in problems in both the Primary Care Triple P intervention group and the control group on most behavioral measures could be a sign of spontaneous recovery. Research on term preschoolers suggests that externalizing behavior generally decreases during preschool age, although internalizing behavior seems to remain relatively stable [33,34]. However, in our study the decrease in emotional and behavioral problems was only present during the first 6 months of the active phase of the trial. A regression to the mean of high emotional and behavioral problem-scores reported during screening is therefore a more plausible explanation. Another explanation that can not be excluded is a Hawthorne effect, in which the decrease in problems reflects the effect of participating in research.

Our study had several limitations. First, we actively recruited families, whereas other studies included families that present themselves to a health care facility. Active recruitment may yield different participants with, for example, different motives to participate, or less urgent child behavior problems. Second, we specifically chose to investigate preterm-born children with gestational ages <32 weeks and term-born children with perinatal asphyxia because, compared to healthy term-born children, they have a twofold risk for emotional and behavioral problems. However, this choice implies that our results are not generalizable to late preterm-born or healthy term-born children. Third, we did not include a post-test only control group. Including a post-test only group would have facilitated the interpretation of our findings that problem behavior decreases in both the intervention and control group, for example by estimating the magnitude of a possible Hawthorne effect. Last, all our measures except for the TRF were parent reported. Therefore, we can only interpret effects as parent reported outcomes, and not actual child behavior.

Study strengths should also be noted. This is the only randomized clinical trial of Primary Care Triple P, and it was conducted independent of Triple P developers. Furthermore, this is the first randomized trial that investigated any level of Triple P exclusively among families with preterm-born children and asphyxiated term-born children.

## Conclusions

This randomized clinical trial demonstrates that the brief parenting intervention Primary Care Triple P is not effective in reducing parent reported child emotional and behavioral problems, in families with a preterm-born child or a term-born child with perinatal asphyxia at preschool age. Furthermore, there was no effect of Primary Care Triple P in terms of parenting styles, parenting stress, and parent perceived child vulnerability. Further research is necessary to create and investigate problem-driven interventions that accommodate the needs of parents of preschool-aged NICU graduates.

## Competing interests

The authors declare that they have no competing interests.

## Authors’ contributions

RS contributed to the design and coordination of the Triple P study, collection, analysis, and interpretation of the data and drafted the manuscript. LW, MUV, and MJ obtained funding for the Triple P study, MJ was the study supervisor. LW contributed to the study design and interpretation of the data. MUV and MJ contributed to the study design, delivery of the intervention, and interpretation of the data. BWW and RV contributed to the delivery of the intervention and collection and interpretation of the data. IT contributed to the analysis and interpretation of the data. CKE and SMT were the attending physicians and contributed to the retrieval of neonatal data. All authors contributed to revisions and approved the final manuscript.

## Pre-publication history

The pre-publication history for this paper can be accessed here:

http://www.biomedcentral.com/1471-2431/13/69/prepub

## Supplementary Material

Additional file 1 CONSORT form. Click here for file
